# Isolation and Screening of Bacteria for Their Diazotrophic Potential and Their Influence on Growth Promotion of Maize Seedlings in Greenhouses

**DOI:** 10.3389/fpls.2015.01225

**Published:** 2016-01-08

**Authors:** Medhin H. Kifle, Mark D. Laing

**Affiliations:** Discipline of Plant Pathology, School of Agricultural, Earth and Environmental Sciences University of KwaZulu-NatalPietermaritzburg, South Africa

**Keywords:** diazotrophs, nitrogen fixation, plant growth promotion, acetylene reduction assay

## Abstract

Poor soil fertility is one of the major constraints for crop production. Nitrogen is the most limiting nutrient for increasing crop productivity. Therefore, there is a need to identify diazotrophic inoculants as an alternative or supplement to N-fertilizers for sustainable agriculture. In the current study, a number of free-living diazotrophic bacteria were isolated from soils collected from maize rhizosphere and from leaves and roots of maize within the KwaZulu-Natal Province, Republic of South Africa. Ninety-two isolates were selected for further screening because they were able to grow on N-free media containing different carbon sources. Isolates that were very slow to grow on N-free media were discarded. The isolates were screened *in vitro* for diazotrophic potential tests for ammonia production and acetylene reduction. Ethylene (C_2_H_4_) production was quantified and ranged from 4 to 73 nmoles of C_2_H_4_h^−1^ culture^−1^. The top 20 isolates were re-screened on maize seedlings, and eight isolates significantly (*P* = 0.001) enhanced some growth parameters of maize above the un-inoculated control. Isolates that showed significant effect on at least two growth parameters were identified at species or genera level. In conclusion, selected diazotrophic isolates may be potentially beneficial but they should be tested more in greenhouse and field conditions with maize to confirm their potential for application as biofertilizers.

## Introduction

Replacement of chemical fertilizers with biofertilizers is an attractive goal for sustainable agriculture. Nitrogen is the macro-nutrient that most frequently limits the growth and productivity of non-leguminous plants (Schepers et al., 1992) and is the most limiting factor in maize production (McCarty and Meisinger, 1997). A number of diazotrophic bacteria were previously found to interact with plants either in the rhizosphere or endophytes. Given the ability of diazotrophs to fix N, some strains may relieve N-deficiencies where there is inadequate application of N fertilizers. The genera *Bacillus, Burkholderia*, and *Enterobacter* are known to penetrate the roots of cereals and grow intercellularly as root endophytes, as well as in the rhizosphere (Reinhold-Hurek and Hurek, 1998; Wakelin and Ryder, 2004).

Some diazotrophic bacteria including those in the genera: *Rhanella, Pantoea, Rhizobium, Pseudomonas, Herbaspirillum, Enterobacter, Brevundimonas*, and *Burkholdera* were found to be associated to maize plants (Montañez et al., 2012). Studies confirmed that the positive effect of some diazotrophic bacterial species on non-leguminous plant yields may not only be due to nitrogen fixation but other mechanisms may also contribute to the growth responses observed in non-leguminous plants (Caballero-Mellado et al., 1992; Vessey, 2003). For example, Abiala et al. (2015) isolated a *Bacillus* strain with multiple growth promoting characterstics from maize rhizosphere. It is also believed that some endophytic diazotrophic bacteria contribute substantial amounts of N to certain graminaceous crops (Barraquio et al., 1997). When some plant seeds were inoculated with diazotrophic bacteria, enhanced seed germination, biomass, increased nitrogen, and chlorophyll content were observed in seedlings (Riggs et al., 2001; Meena et al., 2012). Egamberdiyeva (2007) reported that *Pseudomonas alcaligenes* inoculation stimulated the growth and nutrient uptake of maize. Inoculation of *Bacillus megaterium* and *Bacillus mucilaginous* increased the nutritional assimilation of maize plant (Wu et al., 2005).

*In vitro* screening techniques have been employed to select effective strains of bacteria with multiple plant growth-promotions (Ahmad et al., 2006, 2008; Çakmakçi et al., 2007). Although lack of consistency in correlation between the results obtained *in vitro* and *in vivo* have been reported (Khalid et al., 2004; Yobo et al., 2004), these techniques are simple and efficient for screening large numbers of isolates (Campbell, 1989). Combination of *in vitro* and *in vivo* screening may lead to identification of effective strains for sustainable agriculture. The current study was therefore aimed at isolating diazotrophic bacteria, their partial characterization and for preliminary screening of some growth parameters on maize seedlings in greenhouse experiment.

## Materials and methods

### Bacterial isolation

Diazotrophic bacteria were isolated from rhizospheres, roots and leaves of maize and wheat plants collected from Cedara (Agricultural research collage, Hawick), Greytown, and Ukulinga (University of KwaZulu-Natal research farm, Pietermaritzburg), all within the KwaZulu-Natal province, Republic of South Africa. Roots and leaves were surface sterilized with 3.5% sodium hypochlorite for 5 min and subsequently rinsed three times with sterile distilled water, using a modified protocol of Kloepper et al. (1991). Roots and leaves were cut into pieces and grounded with 10 mL of distilled water. A modified protocol of Döbereiner (1988) (N-free semi-solid media) was used to isolate rhizospheric, rhizoplane, and endophytic diazotrophs. Pure cultures of diazotrophic bacteria were then isolated by serial dilution, and plated onto N-free (NF) media containing of either 20g l^−1^of mannitol, sucrose, or malate as the carbon source; 0.2 g L^−1^ K_2_HPO_4_; 0.2 g L^−1^NaCl; 0.2 g L^−1^; MgSO_4_.7H_2_O; 0.1 g L^−1;^ K_2_SO_4_; 5.0 g L^−1^ CaCO_3_; 20 g L^−1^ agar (Merck) for solid agar media, or with 5 g of agar per liter for a semi-solid media. These bacteria were incubated at 30°C for 4 days.

Soil samples were collected from the rhizosphere of maize and wheat from different sites by uprooting the root system and placing them in plastic bags for transport to the laboratory. They were stored at 4°C for subsequent analysis. Excess soil was shaken off and the soil adhering to the plant roots was collected from each soil sample. Ten grams of each soil sample were transferred to a 250 mL-Erlenmeyer flask containing 90 mL sterile distilled water and shaken at 150 rpm in an orbital shaker incubator for 30 min. Plates with NF medium with mannitol as a carbon source for diazotrophic bacteria were inoculated with 0.1 mL of suspensions obtained from the above dilution procedure (three replicates per dilution). The pH was adjusted to 6.5 using 98% sulphuric acid, and 50% sodium hydroxide. After 5 days of incubation, colonies were transferred onto fresh N-free media, and after 2 days were streaked out onto tryptone soy agar (TSA; Merck) plates. Bacterial isolates were selected by size and shape of colony and by their ability to grow on N-free media. These colonies were sub-cultured onto TSA and incubated at 30°C, purified and stored in 15% glycerol at −80°C.

### Ammonia production test

Ammonia production was analyzed using the qualitative method of Ahmad et al. (2008). Bacterial isolates were tested for the production of ammonia in peptone water. Freshly grown cultures were inoculated in 10 mL peptone water in each tube and incubated for 48–72 h at 28 ± 2°C. Nessler's reagent (0.5 mL) was added in each tube. Development of a brown to yellow color was a positive test for ammonia production.

### Acetylene reduction assay (ARA)

The basis for the assay is the fact that nitrogenase, the enzyme complex in diazotrophic microorganisms that reduces nitrogen to ammonia, also reduces acetylene to ethylene. The diazotrophic nature of all the recovered isolates was determined by ARA. Ethylene was quantified by gas chromatography (GC) and the results were expressed in nano moles of C_2_H_4_ produced h^−1^ culture^−1^. One milliliter of pure culture grown in tryptone soy broth (TSB) for 24 h were inoculated onto 10 mL of nitrogen-free semi-liquid medium, with 0.5% mannitol as a carbon source, solidified by 0.3% gellan gum in 20 mL serum bottles and closed with a red rubber septum (SIGMA-ALDRICH, William Freeman and Co., Ltd.) and incubated for 72 h at 28°C. Bottles that showed bacterial growth were assayed for acetylene reduction. Ten percent of the atmosphere in the bottles was replaced with acetylene (C_2_H_2_), whereas bottles without acetylene were used as the control. After 2 h, at 24°C, 0.25 mL gas samples were taken from each vial and analyzed for amount of ethylene formed using a gas chromatography. These processes were repeated three times from same vial and average were taken for the statistical analysis. A gas chromatograph (Hewlett-Packard 5830A) fitted with a 2–2.1 mm, 80–100 mesh, Poropak R column was used and the oven temperature was adjusted to 70°C. Injection and flame-ionization detector temperatures were adjusted to 150°C. Nitrogen carrier gas flow rate was adjusted to 50 mL min^−1^.

### Source of seeds

Seeds of a white maize cultivar, Mac are Medium Pearl (an open pollinated variety), were purchased from McDonalds Seeds® (MacDonald's Seeds (Ltd).P.O. Box 40, Mkondeni, 3212, Pietermaritzburg, Republic of South Africa) and were used throughout the experiment.

### Inoculum preparation

Bacterial isolates were grown in 100 mL Erlenmeyer flasks each containing a 25 mL tryptic soy broth (TSB; Merck) for 3 d at 28 ± 2°C in a shaker at 150 rpm. Flasks were inoculated with bacteria previously grown in TSA for 48 h. After 3 d bacteria were harvested by centrifugation using a Beckman J2-HS Centrifuge (Beckman Coulter Inc. 4300 N Harbor Boulevard, Box 3100, Fullerton, California, 92834-300, USA) at 9000 rpm for 15 min. The broth was decanted and bacterial pellets were re-suspended in sterile distilled water. Bacterial cells were then counted using a plate dilution technique on TSA plates, and adjusted to a concentration of 10^8^colony forming unit (cfu) mL^−1^ of water.

### Seed treatment

Twenty out of 92 bacterial isolates which produced relatively high C_2_H_4_ levels were selected for further greenhouse screening on maize seedlings. Final cell pellets were diluted with sterile distilled water and cell numbers were adjusted to 2.4 × 10^8^ cfu mL^−1^ for each of the bacterial isolates, as determined by serial dilution and plating. Two grams of a sticker, gum arabic, were dissolved in 100 mL of bacterial suspension, stirred, and allowed to stand for 1 h. This allowed the substance to dissolve and form a homogeneous suspension. Seed coating took place in a plastic bag. The bag was filled with 200 g seeds. The bacterial sticker suspension was added at a rate of 0.1 mL g^−1^ of seeds in order to increase the number of bacteria stuck onto each seed. The bag was closed in such a way to trap air as much as possible. The bag was shaken for 2 min, until all the seeds were uniformly wetted with the sticker suspension. The bag was opened and the seed spreads onto paper towels and air-dried overnight. Treated maize seeds were planted on pine bark artificial growing medium. Composition of the composted pine bark growing medium analyzed by KwaZulu-Natal Agriculture and Environmental Affairs (KZN Agriculture and Environmental Affairs, Private Bag X9059, Pietermaritzburg, 3200, Republic of South Africa) is as follows: 12.39 C (%); 0.11 S (%); 0.43 N (%); 0.61 Ca (%); 0.10 Mg(%); 0.28 K (%); 0.18 P (%); 25.07 Moisture (%); 405.0 Na (mg kg^−1^); 73.0 Zn (mg kg^−1^); 16.7 Cu (mg kg^−1^); 802Mn (mg kg^−1^); 12293 Fe (mg kg^−1^); 7120 Al (mg kg^−1^).

### Measurements of stomatal conductance and chlorophyll content index

Greenhouse measurements of stomatal conductance and leaf chlorophyll were made on 8 weeks old seedlings. Leaf stomatal conductance was measured with a portable porometer (SC-1 Leaf Porometer, Decagon Devices, Inc., 2365 NE Hopkins Court, Pullman, WA 99163, USA). Stomatal conductance readings were made between 9:00 am and 3:00 pm on sunny days on 10 leaves of each pot of nine plants per treatment. Chlorophyll was measured using a portable, handheld device called chlorophyll meter (CCM-200 Plus, Opti-Science Inc., 8 Winn Avenue, Hudson, NH, USA, 03051; that estimates the chlorophyll content of leaves). Measurements were made on eight leaves of each pot of nine plants per treatment.

### DNA extraction and 16S rRNA sequence analysis

One mL of 24 h bacterial culture was centrifuged at 14,000 relative centrifugal force (rcf) for 5 min. The pellet was suspended in 25 μL of (10 mM) Tris and 1 mL of buffer was added and incubated at 60°C for 1 h. One milliliter of CTAB buffer was added and gently mixed. Then the bacterial-buffer suspension were divided into two in 1.5 mL tubes, and 500 μL of chloroform-iso-amyl alcohol was added and mixed gently, and the resultant mixture was centrifuged at 14,000 rcf for 10 min. By avoiding the layer of impurities, 900 μl of clear supernatant was removed as the sample. To this, 600 μl of propan-2-ol was added and refrigerated at −20°C for 1 h. It was centrifuged for 15 min at 4000 rcf and the supernatant was discarded. The pellet were washed with 50 μl of 70% ethanol solution and dried in a laminar flow with lid of the tube being left open for 30 min. The pellet was then suspended in 50 μL of 10 mM Tris (pH 8) or 0.5 X TE buffer. At this point the DNA purity and quality were checked on the nano drop UV spectrophotometer equipment (nano drop 1000, Inqaba Biotech, P.O.Box 1435, Hatfield 0028, Pretoria, South Africa) and a 5 μL sample was run on 0.8% agarose gel [SeakemLE Agarose, Whitehead Scientific (Pty) Ltd www.whitesci.co.za] stained with SYBRSafe nucleic acid stain (Invitrogen) stained with SYBRSafe nucleic acid stain (Invitrogen), with a GeneRuler 1 kb DNA Ladder Plus molecular weight marker (Thermo Fisher Scientific Inc., 81 Wyman street, Waltham, MA 02454, US) to confirm the presence, size and quality of genomic DNA. Once the purity of the DNA was checked, it was sent to the Central Analytic Facility, Stellenbosch University, South Africa for sequencing and BLAST identification. The BLAST identifications were then confirmed by matrix assisted laser desorption ionization-time of flight (Maldi-TOF) classification [Brucker Daltonik Maldi-TOF Biotyper (www.bruker.com)].

### Bruker Daltonik MALDI biotyper classification

Bacterial cultures were sub-cultured on 10% TSA for 24 h at 30°C. A single bacterial colony were taken and placed into a 2 mL eppendorf tube with 300 μL of ultra-pure water, and 900 μL of pure ethanol were added, mixed and the suspension was centrifuged at 14,000 rcf for 2 min. A small pellet of bacterial cells was visible at the bottom of the tube. The liquid was removed, and the pellet was briefly re-spun followed by the removal of residual ethanol. It was then re-suspended in 10 μL of 70% formic acid, and 10 μL of aceto-nitrile was added, and the sample was vortexed briefly. The mixture was centrifuged for 2 min at 14,000 rcf, and the supernatant transferred into a clean micro-tube. The sample to be analyzed was warmed to room temperature, and a 1 μL sample was spotted onto a steel target plate (Bruker Daltonics Inc., Billerica, MA, USA) and gently mixed with 2 μL of matrix solution.

### Experimental design

Maize seedlings treated with bacterial isolates were watered every day with 500 mL nutrient solution containing: 0.11 mL L^−1^ H_3_PO_4_; 0.13 g L^−1^ KOH; 0.14 g L^−1^ K_2_SO_4_; 0.74 g L^−1^ CaCl_2_.2H_2_O; 0.10 g L^−1^ MgSO_4_.7H_2_O; and 0.02 g L^−1^ of micronutrients (MICROPLEX®; Ocean Agriculture (Pty) Ltd, P.O. Box 741, Mulders drift, 1747, South Africa). There were two controls: one was untreated and supplemented with a soluble fertilizer NPK (3:1:3 [38] complete) which was parched from Ocean Agriculture (Pty) Ltd, P.O. Box 741, Mulders drift, 1747, South Africa at a rate of 1 gL^−1^ and the second was untreated control and supplemented with MICROPLEX® nutrient solution. Each treatment consisted of three pots with a top diameter of 200 mm that held 2 kg of composted pine bark. Each pot was seeded with five seeds. The seedlings were thinned to three plants per pot. Pots with each of the 20 isolates were watered every day with an equal amount of a nutrient solution containing phosphorus and potassium of levels adjusted to the full recommended amounts. The experiment was arranged in a randomized complete block design (RCBD), replicated three times. Two months after planting, chlorophyll and stomatal conductance were measured and plants were harvested and dry weight was taken after the biomass was dried in an oven for 72 h at 70°C.

### Statistical analysis

Experiments were repeated twice, unless otherwise stated. Data were analyzed using GenStat executable release 14th edition statistical analysis software. Significant differences between treatments were determined using Duncan's multiple range test at 5% significant level.

## Results

### Isolation and preliminary screening of bacterial diazotrophs

There were differences between diazotrophic isolates, in their ability to grow on semi-solid N-free medium, and using D-mannitol, D-malate or sucrose as carbon sources. Bacterial growth were considered slow when the mass doubling time was longer than 10–12 h and considered well when the mass doubling time was less than 10 h. All these isolates were able to grow well on the N-free semi-liquid medium when sucrose was used as the carbon source, and generated ammonia in peptone water. About 20% of the bacterial isolates grew well on N-free media with D-mannitol, sucrose, or malate as growth substrates. Approximately, 80% of the bacterial isolates showed slow growth on N-free medium with D-mannitol or malate.

### Acetylene reduction assay (ARA)

All the isolates exhibited nitrogenase activity, but the level of activity varied with different isolates (Table 1). Approximately 17% of the isolates produced very little amount C_2_H_4_, no more than the control; 66% of the isolates produced significantly (*P* < 0.001) more C_2_H_4_ compared to the control (N-free semi-liquid medium). The 20 isolates which produced relatively high levels of C_2_H_4_ were further screened in the greenhouse.

**Table 1:** **Nitrogenase activity of the bacterial isolates measured by acetylene reduction assay (ARA)**.

**Isolates**	**nmol of C_2_H_4_h^−1^ culture^−1^**		**Isolates**	**nmol of C_2_H_4_h^−1^ culture^−1^**		**Isolates**	**nmol of C_2_H_4_h^−1^ culture^−1^**	
Broth	0.32	a	M12	21.86	cdefghijklmn	Bt12	29.01	ghijklmnop
Bt14	4.81	ab	StB12	21.91	cdefghijklmn	Bt7	29.46	ghijklmnop
M11	6.20	ab	V1	21.91	cdefghijklmn	StB8	29.87	ghijklmnop
Mr2	6.24	ab	Br2	21.96	cdefghijklmn	G3	31.78	hijklmnopq
SB1	6.46	ab	Bt1	21.96	cdefghijklmn	V3	31.99	ijklmnopq
Mr63	6.51	ab	RB1	21.96	cdefghijklmn	Mr27	32.27	ijklmnopq
Bt10	7.29	abc	Bt15	22.01	cdefghijklmn	V14	32.39	ijklmnopqr
Bt3	7.32	abc	RB6	22.01	cdefghijklmn	Mr57	32.77	jklmnopqr
Mr141	7.61	abc	V12	22.01	cdefghijklmn	Mr34	33.04	klmnopqr
Mr20	7.90	abc	Mr148	22.02	cdefghijklmn	Bt4	33.26	klmnopqr
Mr25	9.10	abcd	RB2	22.06	cdefghijklmn	Bt5	33.49	lmnopqr
Mr8	9.76	abcde	StB7	22.06	cdefghijklmn	V20	33.58	lmnopqr
StB3	10.46	abcde	LB7	22.11	cdefghijklmn	Bt11	33.69	lmnopqr
V15	10.96	abcde	Bt9	22.15	cdefghijklmn	Mr21	33.94	mnopqrs
Mr53	12.26	abcdef	Bt6	22.20	cdefghijklmn	StB13	35.09	nopqrst
Mr35	12.29	abcdef	Bt8	22.20	cdefghijklmn	V10	35.33	nopqrst
E9	12.66	abcdef	V2	22.20	cdefghijklmn	Mr150	35.54	nopqrstu
Mr38	15.40	bcdefg	StB17	22.30	cdefghijklmn	Mr131	37.13	nopqrstu
Mr58	15.96	bcdefg	V6	22.40	cdefghijklmn	StB1	37.23	nopqrstu
Mr6	16.44	bcdefgh	M9	22.41	cdefghijklmn	Mr37	38.82	opqrstuv
V5	17.02	bcdefghi	V7	22.45	cdefghijklmn	a2	42.00	pqrstuv
Mr16	17.22	bcdefghi	LB9	22.55	cdefghijklmn	a3	45.10	qrstuv
V4	17.25	bcdefghij	X	22.80	cdefghijklmn	B1	45.84	qrstuv
Mr19	17.32	bcdefghij	D6	24.19	defghijklmno	a61	46.69	qrstuv
Mr13	17.38	bcdefghij	Mr17	24.19	defghijklmno	a5	47.28	rstuv
Mr7	17.53	bcdefghij	Mr22	24.73	efghijklmno	LB5	48.36	stuv
Mr121	17.76	bcdefghijk	Mr9	25.09	efghijklmno	L1	48.75	tuv
Mr55	18.22	bcdefghijkl	V18	25.20	efghijklmno	a6	49.88	uv
LB2	18.75	bcdefghijklm	V17	27.48	fghijklmnop	StB5	52.37	v
V13	19.05	bcdefghijklm	V19	28.40	ghijklmnop	V16	65.15	w
Bt2	21.86	cdefghijklmn	V11	28.82	ghijklmnop	V9	73.20	w
CV%	30.4							
DMRT	12.035							
Sed	6.101							
*F*-test	9.662							
*P*-value	< 0.001							

*Means with the same letter are not significantly different at P ≤ 0.05. Each treatment was replicated three times (n = 3). Each treatment vial was sampled three times*.

### Effect of diazotrophic inoculation on chlorophyll content index, dry weight and stomatal conductance

Of the 20 diazotrophic isolates, about 50% showed significantly higher (*P* < 0.001) chlorophyll content index, stomatal conductance and dry weight, relative to the untreated control (Table 2). The rest of these isolates had no effect (*P* < 0.05) on the above parameters. Plants of the untreated control showed the lowest stomatal conductance, chlorophyll level and dry weight and plants of the 100% NPK fertilized (NPK control) the highest (Table 2). Isolate StB5 had the highest chlorophyll content index and stomatal conductance but its dry weight was not significantly higher than in the non-inoculated control. The highest dry weight was obtained in the seedlings inoculated with V16 and L1; their chlorophyll content index and stomatal conductance were significantly higher than in control plants.

**Table 2:** **Influence of maize seeds inoculation with diazotrophic bacteria on some growth parameters of 2 months seedlings in greenhouse experiment**.

**Bacterial isolates**	**Chlorophyll content index**		**%Over control**	**Dry weight(g)**		**%Over control**	**Stomatal conductance (m mol m^−2^ s^−1^)**		**%Over control**
Control	4.45	a	–	2.77	a	–	54.2 (1.733)	a	–
Mr21	5.08	ab	14.16	3.63	ab	31.05	121.6 (2.085)	abc	124.35
Mr131	5.24	abc	17.75	3.62	ab	30.69	122.5 (2.086)	abc	126.01
Bt11	5.38	abcd	20.90	3.63	ab	31.05	122.5 (2.087)	abc	126.01
Mr150	5.59	abcde	25.62	4.13	abcd	49.10	142.0 (2.152)	abcd	161.99
StB1	5.83	abcde	31.01	5.17	bcd	86.64	137.7 (2.127)	abc	154.06
StB13	5.95	abcdef	33.71	3.79	abc	36.82	130.3 (2.115)	abc	140.41
Mr37	6.08	abcdef	36.63	3.72	abc	34.30	147.3 (2.165)	abcd	171.77
V20	6.11	abcdef	37.30	5.85	cd	111.19	147.6 (2.169)	abcd	172.32
V10	6.21	abcdefg	39.55	5.79	cd	109.03	134.6 (2.129)	abc	148.34
A61	6.28	bcdefg	41.12	6.23	d	124.91	206.5 (2.295)	cde	281.00
A2	6.37	bcdefg	43.15	5.84	cd	110.83	198.3 (2.274)	cde	265.87
B1	6.52	bcdefg	46.52	3.80	abc	37.18	244.1 (2.383)	de	350.37
A6	6.71	bcdefg	50.79	3.41	ab	23.10	218.1 (2.335)	cde	302.40
A5	7.03	cdefg	57.98	2.42	a	−12.64	188.8 (2.253)	bcde	248.34
A3	7.06	cdefg	58.65	5.26	bcd	89.89	219.3 (2.341)	cde	304.61
LB5	7.11	defg	59.78	5.51	bcd	98.92	203.4 (2.278)	cde	275.28
L1	7.26	efg	63.15	5.85	cd	111.19	206.2 (2.281)	cde	280.44
V16	7.70	fg	73.03	5.99	d	116.25	265.0 (2.423)	e	388.93
V9	7.72	fg	73.48	2.80	a	1.08	253.3 (2.404)	e	367.34
StB5	8.03	g	80.45	3.48	ab	25.63	244.7 (2.386)	de	351.48
NPK	13.62	h	206.07	9.90	e	257.40	517.5 (2.712)	f	854.80
CV%	14			23.9			23.20 (4.2)		
DMRT	1.544			1.833			90.41 (0.198)		
SED	0.765			0.908			43.00 (0.093)		
*P*	< 0.001			< 0.001			< 0.001		

*Means with the same letters in the same column are not significantly different at P < 0.05; values in parentheses are transformed data using log base 10 for the stomatal conductance. Means were calculated from n = 3 for dry weight, n = 24 for chlorophyll content index, and n = 30 for stomatal conductance*.

### Identification of diazotrophic isolates

Comparative analyses of nucleotide sequences of amplified 16S rRNA fragments, using a BLAST approach, revealed that some of the isolates with 99% sequence accuracy. Isolates StB5, A3, A6, B1, and A61 demonstrated this accuracy with *Pseudomonas nitroreducens*, A5 with *Burkholderia* sp., V9 with *Burkholderia ambifaria*, and A2 with *Klebsiella variicola*. Isolate L1 showed 98% similarity with *Enterobacter cloacae*; V16 (97%) with *B. megaterium* and LB5 (97%) with *Pantoea ananatis* (Table 3). With laser desorption ionization-time of flight (Maldi-TOF) classification system, a score of ≥2.000 indicates species level identification, a score of 1.700–1.999 indicates identification to the genus level, and a score of < 1.700 is interpreted as no identification. Isolates StB5, A3, A6, and A61 were identified as *Pseudomonas* sp with MALDI-TOF scores of 1.98, 1.90, 1.96, and 1.88, respectively. Isolate B1 (2.03) as *P. nitroreducens*, V9 (2.46) as *Burkholderia ambiferia* and A5 (1.86) were identified as *Burkholderia* sp, isolate L1 (2.33) as *E. cloacae*, isolate V16 (1.72) as *Bacillus* sp, A2 (2.24) as *K. variicola* and isolate LB5 (2.27) as *Pantoea ananatis*. An independent identification of these isolates, based on 16S rRNA gene and Maldi-TOF biotyper, confirmed their identity to species and genera level.

**Table 3:** **Affiliation of the isolates in the GenBank and the identification of the closest type strain based on the 16S rRNA gene sequencing and Bruker Daltonik MALDI-TOF Biotyper classification**.

**Isolates**	**16S rRNA similarities (highest match)**	**BrukerMALDI biotype (highest score)**
V16	*Bacillus megaterium* Strain As-30 (97%)	*Bacillus* sp. (1.722)
A5	*Burkholderia* sp. IBP-VNS127 (99%)	*Burkholderia* sp. (1.867)
V9	*Burkholderia ambifaria* (99%)	*Burkholderia ambifaria* (2.462)
L1	*Enterobacter cloacae* Strain G35-1(98%)	*Enterobacter cloacae (2.327)*
A2	*Klebsiella variicola* (99%)	*Klebsiella variicola* (2.243)
LB5	*Pantoea ananatis* (97%)	*Pantoea ananatis* (2.268)
A3	*Pseudomonas nitroreducens* Strain R5-791 (99%)	*Pseudomonas* sp. (1.901)
A6	*Pseudomonas nitroreducens* (99%)	*Pseudomonas* sp. (1.96)
B1	*Pseudomonas nitroreducens* (99%)	*Pseudomonas nitroreducens* (2.034)
StB5	*Pseudomonas nitroreducens* Strain R5-791 (99%)	*Pseudomonas* sp. (1.989)
A61	*Pseudomonas nitroreducens* (99%)	*Pseudomonas* sp. (1.882)

## Discussion

Isolation and screening for potential diazotrophic bacteria are crucial steps in research on biofertilizers. In order to discover efficient nitrogen fixing bacteria, there is a need to develop simple, inexpensive and quick procedures with repeatable and reliable results (Döbereiner, 1988). Such as an *in vitro* screening procedure (growth on N-free semi-solid media, ARA, and the ammonia production test) and the combinations of which provides rapid and repeatable results. All the isolates proved to be nitrogen fixers. Though it is difficult to compare the nitrogenase activity of bacterial strains studied in this work with the results obtained by others, a study by Różycki et al. (1999) showed similar results of nitrogenase activity by some of diazotrophic isolates belonging to the genera *Pseudomonas* and *Bacillus*.

About 50% of the best isolates used in this study were identified as *Pseudomonas*. In another study, the genera of *Pseudomonas* and *Enterobacter* were reported to be dominant in maize cultivars (Rodríguez-Blanco et al., 2015). Moreover, Berge et al. (1991) reported that an *E. cloacae was* the most abundant diazotrophic bacterium in the rhizosphere of maize-growing soils in France. *Klebsiella* and *Burkholderia* were also dominant genera both in roots and rhizosphere of maize (Arruda et al., 2013). Estrada et al. (2002) isolated a strain of endophytic, N_2_-fixing *Burkholderia* sp. associated with maize in Mexico. Similarly, Perin et al. (2006) isolated *Burkholderia* sp. from the rhizosphere of maize. The predominance of these genera both in the soil and in the root zone may be due to low nutritional requirements, its capacity to utilize numerous complex of organic substrates (Krotzky and Werner, 1987) and high tolerance to low pH (Eckford et al., 2002).

In this study, some of the bacterial isolates with high nitrogenase activity were found to improve chlorophyll content, stomatal conductance and dry weight in maize. All the tested isolates showed increases (124.4–351.5%) in stomatal conductance, nine isolates showed (86.6–24.9%) increases in dry weight, and 11 of the isolates (41.12–80.45%) increases in chlorophyll content index over the control. Diazotrophic microbes isolated either from soil or endophytes increased sweet corn biomass both in greenhouse and field trials (Mehnaz and Lazarovits, 2006; Mehnaz et al., 2010). In another study, interaction of diazotrophic plant growth promoting rhizobacteria with sugar cane, cotton, wheat, rice, and maize significantly increased the vegetative growth and grain yield (Kennedy et al., 2004). Likewise, maize plants inoculated with endophytic diazotrophs such as (*Herbaspirillum* sp, *Psuedomonas* sp., and *Burkholderia vietnamienis*) gained greater early biomass and higher rates of net CO_2_ assimilation than the un-inoculated control (Knoth et al., 2013). Furthermore, dry matter of maize increased by 25–54% after inoculation with strains *Achromobacter* sp., *Burkholdria* sp. and *Arthrobacter* sp. (Arruda et al., 2013). In this study, inoculation of maize seeds with *Burkholderia ambifaria* (V9), *Bacillus* sp. (V16)*, P. nitroreducens* (B1), Pseudomonas spp. (A3, A6, A61, StB5), *E. cloacae (L1), P. ananatis (LB5), and Klebsiella variicola (A2*) enhanced stomatal conductance and chlorophyll content index compared to the control. The increases could be a result of increased leaf N through nitrogen fixation by these diazotrophs. Nitrogen fixed by diazotrophic bacteria may not fully replace chemical fertilizers however; use of diazotrophic bacterial inoculants may be an important factor for sustainable agriculture.

## Conclusion

It can be concluded that the selected diazotrophic isolates may be potentially beneficial biofertilizers and should be tested more in field conditions to confirm their potential to use as biofertilizers inoculants.

### Conflict of interest statement

The authors declare that the research was conducted in the absence of any commercial or financial relationships that could be construed as a potential conflict of interest.
